# Mobile App for Treatment of Stress Urinary Incontinence: A Cost-Effectiveness Analysis

**DOI:** 10.2196/jmir.7383

**Published:** 2017-05-08

**Authors:** Malin Sjöström, Lars Lindholm, Eva Samuelsson

**Affiliations:** ^1^ Department of Public Health and Clinical Medicine Umeå University Umeå Sweden

**Keywords:** mobile application, pelvic floor, urinary incontinence, stress, self care, cost-benefit analysis

## Abstract

**Background:**

Mobile apps can increase access to care, facilitate self-management, and improve adherence to treatment. Stress urinary incontinence (SUI) affects 10-35% of women and, currently, an app with instructions for pelvic floor muscle training (PFMT) is available as first-line treatment. A previous randomized controlled study demonstrated that the app benefitted symptom severity and quality of life (QoL); in this study we investigate the cost-effectiveness of the app.

**Objective:**

The objective of this study was to evaluate the health economy of the app for treating SUI.

**Methods:**

This deterministic cost-utility analysis, with a 1-year societal perspective, compared the app treatment with no treatment. Health economic data were collected alongside a randomized controlled trial performed in Sweden from March 2013 to October 2014. This study included 123 community-dwelling women participants of 18 years and above, with stress urinary incontinence ≥1 time per week. Participants were self-assessed with validated questionnaires and 2-day leakage diaries, and then randomized to 3 months of treatment (app group, n=62) or no treatment (controls, n=61). The app focused on pelvic floor muscle training, prescribed 3 times daily. We continuously registered treatment delivery costs. Data were collected on each participant’s training time, incontinence aids, and laundry at baseline and at a 3-month follow-up. We measured quality of life with the International Consultation on Incontinence Modular Questionnaire on Lower Urinary Tract Symptoms and Quality of Life, and calculated the quality-adjusted life years (QALYs) gained. Data from the 3-month follow-up were extrapolated to 1 year for the calculations. Our main outcome was the incremental cost-effectiveness ratios compared between app and control groups. One-way and multiway sensitivity analyses were performed.

**Results:**

The mean age of participants was 44.7 years (SD 9.4). Annual costs were €547.0 for the app group and €482.4 for the control group. Annual gains in quality-adjusted life years for app and control groups were 0.0101 and 0.0016, respectively. Compared with controls, the extra cost per quality-adjusted life year for the app group ranged from −€2425.7 to €14,870.6, which indicated greater gains in quality-adjusted life years at similar or slightly higher cost.

**Conclusions:**

The app for treating stress urinary incontinence is a new, cost-effective, first-line treatment with potential for increasing access to care in a sustainable way for this patient group.

## Introduction

One possible way to meet the future demands in the health care sector could be to empower patients by increasing self-management with mHealth [1]. Worldwide, there are approximately 5 billion mobile phone subscribers, and the smartphones are constant companions for many individuals [2]. The App Store and Google Play websites offer around 100,000 health apps, but few have been scientifically evaluated [3]. Mobile health apps could facilitate self-management and adherence to treatment; in addition, they could increase access to care for individuals with limited access or for those unwilling to seek ordinary health care [4].

Stress urinary incontinence (SUI), that is, urine leakage upon sneezing, coughing, or exertion [5], affects 10-35% of women [6,7]. This condition is suited to self-management. The diagnosis is based on patient-reported measures and does not require a physical examination [8]. The first-line treatment is pelvic floor muscle training (PFMT), which is safe, effective [8-10], and can be completed without health care personnel supervision [8]. Although SUI can decrease quality of life (QoL) [11], only around 20% of individuals seek care [12]. In some cases, the leakage is not considered as a major problem, but in other cases, the patient is too embarrassed to seek care [13]. Our research group has developed the mobile app, Tät, which serves as a first-line treatment for SUI, based on self-management. This app provides information and instructions for PFMT [14].

One concern in deciding the treatments to be delivered in the health care systems is the cost. One common way to evaluate cost is the cost-utility analysis, which compares the costs and effects of at least two treatment alternatives. This analysis allows comparison of diverse interventions [15]. Costs can be considered either from a health care perspective, which only includes costs borne by the health care system, or from a societal perspective, which includes other costs. Currently, the former perspective is recommended in the United Kingdom by the National Institute for Health and Clinical Exellence (NICE) [16], and the latter is recommended in the United States [17] and Sweden [18]. The utility of the treatment is defined as the added time gained with an improved QoL, calculated as quality-adjusted life years (QALYs).

In this study, we performed a cost-utility analysis of SUI treatment with the app, Tät, compared with no treatment.

## Methods

### Design

This deterministic cost-utility analysis had a 1-year societal perspective. It was performed according to the principles outlined by Drummond et al [15].

### Population

We collected data for this analysis alongside a randomized controlled trial on SUI treatment with the Tät app. The trial was registered at clinicaltrials.gov (ID: NCT01848938), and the trial results were described in detail elsewhere [14].

Briefly, we recruited community-dwelling women, aged 18 years and above, with SUI of once or more than once a week, via our website. Interested women completed an online screening questionnaire. When they met the study criteria, we sent them a letter of information, a form to provide informed consent, and a 2-day leakage diary. After returning these, they completed a Web-based questionnaire that provided their background characteristics, medical history, symptom severity, and QoL. Exclusion criteria were ongoing pregnancy, maximum voiding volume <0.3 L, macroscopic hematuria, irregular menstrual bleeding, difficulty passing urine, previous incontinence surgery, previous or present malignancy in the lower abdomen, severe psychiatric disorder, or impaired mobility or sensibility in the legs or lower abdomen.

We consecutively randomized eligible women to either three months of treatment with the app (app group, n=62), or no treatment (control group, n=61). The app Tät contained information on SUI, provided a PFMT program, with 6 basic and 6 advanced levels, and it prescribed PFMT 3 times daily during treatment. At the end of treatment, the instructions were to continue PFMT 2 or 3 times per week for maintenance training [19]. The control group received no intervention. After the 3-month follow-up, we offered the participants in the control group the app, on an optional basis. We collected 3-month follow-up data with a Web-based questionnaire. There was no face-to-face contact with the participants at any time.

### Symptom Severity

We measured symptom severity at baseline and at 3 months, with the validated [20] and recommended [8,10,21] questionnaire, the International Consultation on Incontinence Modular Questionnaire on Urinary Incontinence, Short Form (ICIQ-UI SF). It contained 3 items such as frequency, amount of leakage, and overall QoL impact. The total score ranged from 0 to 21, with higher scores indicating greater severity. The total scores were used to categorize the severity of the condition (1-5: slight; 6-12: moderate; 13-18: severe; and 19-21: very severe) [22]. After 3 months, the app group reported clinically relevant and significantly greater improvements in symptoms compared with the control group [14].

### Costs

We evaluated costs from a 1-year societal perspective. Costs included the cost of mailing the 2-day leakage diary, and the estimated time spent by our study administrator in emailing each participant the link to the Web-based questionnaire. The cost for our study administrator’s time was calculated based on her gross hourly wage. We did not include costs for the app development because these are one-time costs and are comparable with, for example, the costs for basic education of health care personnel; these are costs which are normally not included in health economic analyses. No other costs for the delivery of treatment were identified.

We collected baseline and follow-up data on the use of incontinence aids and any extra laundry due to leakage. In a previous study, we collected data on the different types of incontinence aids (large, medium, or small) used by women with SUI [23]. We then calculated a mean price per unit, based on the prices for incontinence aids listed on the website of a large pharmacy brand (Apoteket). The price for laundry was derived from the literature [24].

When a societal perspective is applied, an estimate of the cost for the individual’s time should be included in the health economic analysis [14]. At the 3-month follow-up, participants estimated how much time they had spent on PFMT during the last 4 weeks. We used this estimate to calculate the PFMT performed during the treatment period. To estimate PFMT for the entire year for the app group, we assumed that the participants would follow the prescription for maintaining PFMT over the remaining part of the year and we adjusted the time spent on PFMT accordingly. For the control group, we assumed the time spent on PFMT would remain constant throughout the year. To estimate the cost for each participant’s time, we calculated the gross hourly wages for women with the same educational level in Sweden [25], a method which is commonly used [14].

For all other costs, we assumed that costs measured at the 3-month follow-up would remain constant throughout the year. We added up all the costs, and the sum represented the total societal cost. All costs are given in euro, and they were based on the 2013 year-end prices. At that time, the exchange rate for 1 EUR was 8.94 SEK (Swedish krona).

### Quality of life, Utility Weights, and QALYs

To evaluate QoL, we used the validated [26-28] and recommended [8,10,24] condition-specific questionnaire, the International Consultation on Incontinence Modular Questionnaire on Lower Urinary Tract Symptoms and Quality of Life (ICIQ-LUTSqol). This questionnaire contained 19 items on aspects of everyday life that might be influenced by urinary leakage, such as travel, work, meetings with family and friends, exercise, sexual performance, mood, energy, and sleep. Items are scored 1-4 (1: not at all or never; 2: slightly or sometimes; 3: moderately or often; and 4: a lot or all the time). The overall score ranged from 19 to 76, with higher scores indicating more impact. The questionnaire, was derived from the Kings Health Questionnaire [26], which is widely used in health economic analyses; both questionnaires used the same method for calculating QALY [29].

We based our QALY calculations on data from the ICIQ-LUTSqol, and we applied a preference-based index derived by Brazier et al [29], which incorporates 9 of the 19 items into a “health state” classification. We used the algorithm of this index to translate the health state classification into a utility weight, which ranged from 0 (worst imaginable health state) to 1 (best imaginable health state). We assumed that the utility weight calculated from 3-month follow-up data would remain stable for the remainder of the year in both groups.

### Main Outcome

**Figure 1:**
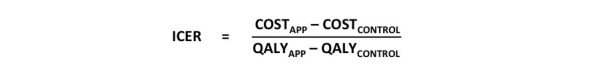
Equation for calculation of the incremental cost-effectiveness ratio (ICER) using costs and quality-adjusted life-years (QALYs) for the app and control group respectively.

Our main outcome was the incremental cost-effectiveness ratio (ICER), defined as the difference in cost-effectiveness between the app group and the control group. We calculated the ICER as presented in Figure 1.

### Statistics

For group comparisons at baseline, we used the Student *t* test for continuous variables, the chi-square test for categorical variables, and the Mann-Whitney *U* test for ordinal variables. For analyses of treatment effects within each group, we used paired *t* test. For comparisons of treatment effects between groups, we used a linear mixed-model analysis, which incorporated all available data for the outcomes of symptom severity and QoL. The utility weights are expressed as the mean value with a 95% CI. Costs were assumed to change linearly, and QALYs were calculated based on an “area-under-the-curve.”

*P* values <.05 were considered statistically significant. We collected and analyzed data in SPSS for Mac, version 23.0 (IBM) and in Excel for Mac, version 14.6.3 (Microsoft Corporation).

### Sensitivity Analysis

We considered the fact that PFMT could be performed while doing other things and that laundry caused by leakage could be washed with other garments. Therefore, we performed a one-way sensitivity analysis by varying input data on the time spent on PFMT, the time spent on laundry, and the cost of laundry, one at a time, to test the potential impact of these uncertainties. In addition, we performed a multiway analysis that incorporated all three variables.

### Ethics

The Regional Ethical Review Board, Umeå University, approved of the study (number 2012-325-31M). All participants gave informed consent.

## Results

### Study Population

We performed this study in Sweden, from March 2013 to October 2014. We randomized 123 participants to receive the app (app group, n=62) or with no treatment (control group, n=61). Baseline characteristics, including age, education, symptom severity, and baseline QoL scores did not differ significantly between groups (Table 1).

At follow-up, we had lost one participant from each group. In addition, in the app group, we were missing outcome data on the ICIQ-LUTSqol for 3 participants.

### Costs

The total assessment cost per participant was €6.4. The app group had higher total costs than the control group, mainly due to the extra time spent on PFMT. The total annual cost per participant in each group is presented in Table 2.

**Table 1:** Baseline characteristics of study participants.

Variable		App group (n=62)	Control group (n=61)
Age, mean years (SD^a^)		44.8 (9.7)	44.7 (9.1)
University education ≥3 years, n (%)		52 (84)	46 (75)
BMI^b^, mean kg/m^2^ (SD)		24.0 (4.1)	24.5 (4.4)
Daily smoker, n (%)		2 (3)	3 (5)
**Symptom severity, n (%)^c^**			
	Slight	3 (5)	0 (0)
	Moderate	36 (58)	42 (69)
	Severe	23 (37)	19 (31)
Overall score ICIQ-UI SF^c^, mean (SD)		11.1 (3.0)	11.0 (2.6)
Overall score ICIQ-LUTSqol^d^, mean(SD)		34.1 (6.1)	34.8 (6.1)

^a^SD: standard deviation.

^b^BMI: body mass index.

^c^Based on overall score from the International Consultation on Incontinence Modular Questionnaire on Urinary Incontinence, Short Form (ICIQ-UI SF).

^d^ICIQ-LUTSqol: International Consultation on Incontinence Modular Questionnaire on Lower Urinary Tract Symptoms, Quality of Life.

**Table 2:** Costs per participant included in a cost-effectiveness analysis with a 1-year societal perspective, for App group versus Control group in women with stress urinary incontinence.

Variable		Price per unit^a^	Amount used		Cost	
			App group	Control group	App group	Control group
Assessment		6.4	1	1	6.4	6.4
Treatment delivery		0	-	-	0	0
**Participant costs**						
	Participant's time for PFMT^b,c^, mean (h)	29.61	15.66	9.91	463.7	293.4
	Participant's time for laundry^c^, mean (h)	29.61	1.30	3.38	38.5	100.1
	Incontinence aids^d^, mean (n)	0.134	114.40	169.60	15.4	22.7
	Extra laundry loads^e^, mean (n)	2.21	10.40	27.04	23.0	59.8
Total cost					547.0	482.4

^a^Prices are in euro (€), based on the 2013 year-end prices. Exchange rate was 1 EUR=8.94 SEK.

^b^PFMT: pelvic floor muscle training.

^c^Based on 2013 mean income for Swedish women with a similar educational level.

^d^Based on mean consumption at baseline (prices acquired from Apoteket).

^e^Data from the literature [24].

### Quality of Life, Utility Weights, and QALYs

In the app group, there was significant improvement in QoL at follow-up (mean ICIQ-LUTSqol reduction: 4.8, 95% CI 3.4-6.2). In contrast, the control group did not display a significant reduction in scores (mean ICIQ-LUTSqol reduction: 0.7, 95% CI −0.5 to 1.8). The difference between groups was highly significant (*P*<.001).

**Figure 2:**
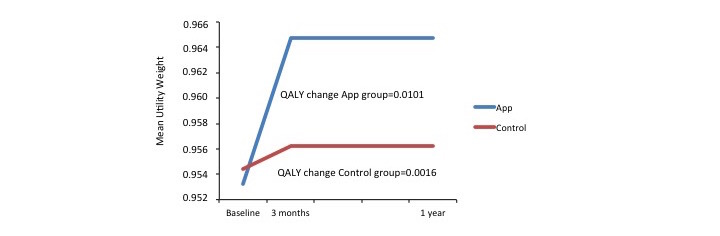
Changes in utility weights reflect gains in quality-adjusted life years (QALYs). Assessments were recorded at baseline, and at 3-month follow-ups, and estimated at one-year, for individuals that received either the app treatment (top, blue line) or no treatment (bottom, red line) for stress urinary incontinence.

The utility weights and QALY changes for each group are presented in Figure 2. The QALYs gained corresponded to an extra 3.9 days in the best imaginable health state for the app group, and only 0.6 days for the control group.

### Main Outcome and Sensitivity Analysis

In Table 3, we illustrate the ICERs for the base case and the sensitivity analysis. In all the analyses, except one (participant’s time for PFMT halved), the costs in the app group were slightly higher than costs in the control group. However, in all cases, the app treatment was more effective compared with no treatment or control group.

**Table 3:** Incremental cost-effectiveness ratios (ICERs) for the app group versus the control group, including the base case and a sensitivity analysis.

Group			Total cost (€^a^)	QALY^b^-gain	∆ Cost (€)	∆ QALY-gain	ICER^c^
Base case							
		Control group	482.4	0.00158			
		App group	547.0	0.01006			
		App group vs control group			64.6	0.00849	7615.5
Sensitivity analysis							
	**One-way: participant’s time for PFMT^d^halved**						
		Control group	335.7	0.00158			
		App group	315.1	0.01006			
		App group vs control group			−20.6	0.00849	−2425.7
	**One-way: cost for laundry halved**						
		Control group	452.4	0.00158			
		App group	535.4	0.01006			
		App group vs control group			83.1	0.00849	9785.5
	**One-way: participant’s time for laundry not included**						
		Control group	382.3	0.00158			
		App group	508.5	0.01006			
		App group vs control group			126.2	0.00849	14870.6
	**Multiway: participant’s time for PFMT and cost for laundry halved, participant's time for laundry not included**						
		Control group	205.7	0.00158			
		App group	265.1	0.01006			
		App group vs control group			59.4	0.00849	6999.5

^a^€ refers to euro at 2013 year-end price.

^b^QALY: quality-adjusted life years.

^c^ICER: ∆ Cost/∆ QALY-gain.

^d^PFMT: pelvic floor muscle training.

## Discussion

### Principal Findings

In this health economic evaluation, we demonstrated that SUI self-management with a mobile app that provided information and instructions for PFMT was a cost-effective first-line treatment alternative, compared with a control group that received no treatment. The results were consistent and stable in different scenarios with varying costs.

### Strengths and Limitations With the Study

This study had several strengths. The calculations were based on known costs and on data collected directly from the participants. Our research group had previous experience with non–face-to-face SUI treatment; there were no disruptions or major technical problems during the study and the loss to follow-up was low. We applied existing guidelines and used validated and recommended outcomes. The diagnosis of SUI was well substantiated and the population was clinically relevant because the vast majority (120/123, 97.6%) of participants had moderate to severe symptoms and actively sought treatment.

This study also had some limitations. One was the relatively low number of participants (n=123), which increases the uncertainty of the data and might have affected the results. Another was that we did not have 1-year follow-up data. Instead, we assumed that costs and utility weights measured at the 3-month follow-up would remain constant over the year. This assumption was based on our previous study of long-term effects of Internet-based PFMT for SUI [30], where improvements achieved after 3 months of treatment were maintained after 1 and 2 years. We had no reason to believe that the outcome of the current app treatment would be different from that of the previous study. Moreover, although the no-treatment alternative was plausible, given the fact that only around 20% of affected women seek care [12], it would have been interesting to compare outcomes between the app and a care-as-usual alternative. However, care-as-usual varies substantially, because there is no gold standard for SUI treatment. Another limitation was that our population had a higher educational level (98/123, 79.7% had ≥3 years of university education) than the general population of Swedish women (≈30% of women aged 25-44 years have ≥3 years of university education) [31]. However, there are no indications that the educational level might affect the ability to perform PFMT [32].

### Strengths and Weaknesses Compared With the Literature

The total cost per participant was higher in the app group (€547.0) than that in the control group (€482.4). Although savings on laundry and incontinence aids were larger in the app group, participants in this group spent more time on PFMT. However, our estimation of the cost for participant time might have been somewhat biased, due to the relatively high educational level of our participants, compared with that of the general population. Nevertheless, the results were consistent in all tested scenarios, and the costs were comparable with those reported in other studies on conservative SUI treatments. For example, in a previous study, we compared Internet-based programs and postal-treatment programs for PFMT, where we found that the total costs were €596.5 and €596.2, respectively [20]. Moreover, in a Dutch study on a care-as-usual SUI treatment, the total cost was €453 (including productivity losses, travel costs, patient out-of-pocket costs, and health care costs, but not time for PFMT) [33].

In this study, we most likely overestimated the QALY gains in the control group due to the fact that we considered it significant in the incremental analysis despite of controls not showing a significant improvement in QoL. The app-group gain in QALY (0.0101) might seem small, but it was comparable to QALY gains observed in other studies on SUI treatment. In a primary care setting, Albers-Heitner et al [33] reported incremental QALY gains of 0.01-0.02, when intense PFMT was performed under guidance of a specialist nurse and compared with a general practitioner (GP) care-as-usual alternative. Arlandis-Guzman et al [34] reported QALY gains of 0.01014, 0.00846, and 0.00957, with the antimuscarinic drugs fesoterodine, tolterodine, and solifenacin, respectively. However, second-line treatments with sling surgery could produce larger QALY gains (0.0504) [35]. Nevertheless, although the QALY gains with conservative treatments are low, in sheer numbers, the patient group that can potentially benefit from treatment is large; thus, the attainable total QALY gain is substantial.

We estimated that the extra cost per QALY for the app treatment was €7615.5, and the sensitivity analyses indicated a potential range of −€2425.7 to €14,870.6. In one of the scenarios tested (time for PFMT halved), the negative ICER value implied that, compared with doing nothing for this group of patients, the app treatment could increase the QoL for the individual at a reduced cost for the society. In the other scenarios, QALY gains were larger in the app group, but at greater cost, compared with the control group. The affordability of an additional cost per QALY depends on the willingness to pay for a more effective treatment, which might differ in different countries. In the United Kingdom, interventions with ICERs ≤€16,500-25,000 (£20,000–30,000) are typically recommended by NICE [16], and in US, those with ICERs of ≤€36,500 ($50,000) are usually recommended [36,37]. In Sweden, incremental costs of ≤€11,200 (100,000 SEK) are considered low, and incremental costs of ≥€60,000 (500,000 SEK) are considered high [38]. Data are scarce on the cost effectiveness of other health apps, due to the limited number of studies conducted.

We did not calculate an ICER from a health care perspective, because the assessment cost was the same (€6.3) in both the groups, and no costs could be identified for the delivery of treatment, that is, ∆ Cost=0. When the app is implemented outside a study setting, the parameters will change. For example, additional costs for updates, bug fixes, and technical support must be taken into account. We estimate that an IT technician would require approximately 4 h per month for this maintenance, but on the other hand, as the number of users increases, the cost per person will diminish. Furthermore, the total cost for the health care system to deliver an app treatment is likely to be low compared with face-to-face treatment. For example, in Sweden, the estimated cost for a GP consultation was €173 [39]; in United Kingdom, the estimated cost for 3 months of PFMT under supervision of a trained nurse was €158 to €293 [40].

### Future Research and Clinical Implications

The Tät app has been released free of charge in both Swedish and English. Although the cost per participant will decrease as the number of users increases, the effects might decline outside the study setting. We are currently continuing to follow the effects reported by users. In addition, the long-term effects of the app need to be established, and the app should also be evaluated as a possible complement to other treatments. Future perspectives include developing the app for treating other types of urinary incontinence.

SUI treatment with the Tät app will not suit all women, but it offers a cost-effective first-line treatment to many women. To our knowledge, this study was the first to evaluate the cost effectiveness of an app treatment for a common health condition. Modern health care systems face many challenges, and it is important for clinicians to deliver care in sustainable ways. The development of self-management apps could be a feasible way to deliver high-quality care in a cost effective, affordable manner to large patient groups. It could also be a way to provide treatment to women with limited access to care, for example, women in low or middle-income countries. While to adding value to the individual patient, these apps could reduce the need for support from primary care, and thus, those resources could be conserved for individuals with explicit needs.

### Conclusion

Self-management of SUI with an app for PFMT is a cost-effective first-line treatment alternative.

## Abbreviations

GP: general practitioner
ICER: incremental cost-effectiveness ratio
ICIQ-LUTSqol: International Consultation on Incontinence Modular Questionnaire on Lower Urinary Tract Symptoms and Quality of Life
ICIQ-UI SF: International Consultation on Incontinence Modular Questionnaire on Urinary Incontinence, Short Form
NICE: National Institute for Health and Clinical Exellence
PFMT: pelvic floor muscle training
QALY: quality-adjusted life year
QoL: quality of life
SUI: stress urinary incontinence
